# A National Strategy for Preventing Substance and Opioid Use Disorders Through Evidence-Based Prevention Programming that Fosters Healthy Outcomes in Our Youth

**DOI:** 10.1007/s10567-022-00420-5

**Published:** 2022-12-21

**Authors:** Diana H. Fishbein, Zili Sloboda

**Affiliations:** 1 Present Address: Frank Porter Graham Child Development Institute, University of North Carolina-Chapel Hill, 105 Smith Level Road, Chapel Hill, NC 27599 USA; 2 The Pennsylvania State University, State College, PA USA; 3 National Prevention Science Coalition to Improve Lives, Oakland, CA USA; 4 Applied Prevention Science International, Ontario, OH USA

**Keywords:** Prevention science, Public policy, Opioid use disorder, Comprehensive prevention system, National strategy, Evidence-based

## Abstract

The recently released National Drug Control Strategy (2022) from the White House Office of National Drug Control Policy (ONDCP) lays out a comprehensive plan to, not only enhance access to treatment and increase harm reduction strategies, but also increase implementation of evidence-based prevention programming at the community level. Furthermore, the Strategy provides a framework for enhancing our national data systems to inform policy and to evaluate all components of the plan. However, not only are there several missing components to the Strategy that would assure its success, but there is a lack of structure to support a national comprehensive service delivery system that is informed by epidemiological data, and trains and credentials those delivering evidence-based prevention, treatment, and harm reduction/public health interventions within community settings. This paper provides recommendations for the establishment of such a structure with an emphasis on prevention. Systematically addressing conditions known to increase liability for behavioral problems among vulnerable populations and building supportive environments are strategies consistently found to avert trajectories away from substance use in general and substance use disorders (SUD) in particular. Investments in this approach are expected to result in significantly lower rates of SUD in current and subsequent generations of youth and, therefore, will reduce the burden on our communities in terms of lowered social and health systems involvement, treatment needs, and productivity. A national strategy, based on strong scientific evidence, is presented to implement public health policies and prevention services. These strategies work by improving child development, supporting families, enhancing school experiences, and cultivating positive environmental conditions.

## Introduction

The United States (US) finds itself in a multitude of epidemics – more aptly referred to as a “syndemic”. For several years now, the nation has witnessed an unprecedented rise in opioid overdoses from both nonmedical use of prescription pain relievers and opioids accessed through street sources (Schnell & Currie, 2018), resulting in a 200% increase in opioid-related deaths in one decade. The opioid crisis claims more lives than from car crashes, gun violence, or murders altogether on an annual basis. And from the start of the COVID-19 pandemic, rates of opioid use and overdose deaths reached unimaginable proportions (CDC, 2021a). In a 12-month period between 2020 and 2021, the U.S. lost more than 100,000 people to overdose deaths (CDC, 2021b).

Families and communities across the US have been devastated by the ready availability of dangerous synthetic opioids such as fentanyl, at a time when formal and informal protective support networks have been disrupted by the pandemic. Furthermore smoking, illicit and unprescribed drug use, and alcohol use rank as the second, eighth, and twelfth contributors, respectively, to mortality in the U.S., totaling close to 700,000 deaths per year (Ritchie & Roser, 2019). These estimates do not include the additional contribution that these substances make to the country’s morbidity and social and economic burden on families and communities.

What is not widely known is that prevention science offers solutions to the ongoing growth of this crisis. Over the past 30 years, a vast amount of research has enhanced our knowledge about the factors and processes leading to misuse of substances resulting in a broad array of evidence-based interventions and strategies (e.g., Biglan et al., 2020; Kuklinski et al., 2021; Van Ryzin et al., 2018). Systematically addressing conditions known to increase risk for behavioral problems in vulnerable populations and building or reinforcing conditions that foster resiliency are strategies consistently found to prevent substance use disorder (SUD) in general, and opioid use disorder (OUD) in particular. Investments in this approach will result in significantly fewer problems with substance use and other related behavioral and mental health issues in our current and subsequent generations of youth. In effect, prevention strategies are expected to be highly cost effective in terms of lowered levels of social and health service involvement, the need for treatment, and productivity (NIDA, 2022).

Based on a strong scientific foundation, this paper outlines a strategy for the implementation of public health policies and prevention services that address opioid use and, in effect, the use and abuse of all psychoactive substances that negatively impact individuals, their families, and communities. Such an approach is only effective when integrated into a comprehensive national service delivery system, based on an assessment of need, provided at the community level, and supported by a monitoring data structure.

## Substance Use Is a Public Health Problem and Why It Matters

The unprescribed use of substances that impact the central and peripheral nervous systems (brain, spinal cord, and autonomic nervous system) can have harmful health and lifestyle consequences. Unprescribed substance use differs from routine use of medications prescribed by health care professionals in that phased efficacy and effectiveness trials have demonstrated the ability of prescription medications to remediate specific medical conditions. In addition, medications are manufactured under strict, rigorously controlled conditions to assure regulated consistency by the Food and Drug Administration (FDA). Ignoring the health consequences of unsupervised or unmonitored use poses a risk to the user’s condition and survival, as well as to other people in their sphere and the larger community. For children and adolescents, the principal concern is that these substances impact the developing brain and other organs (Hamid et al., 2022; Lees et al., 2021), and increase risk for developing a SUD (Chen et al., 2009). For both adolescents and adults, certain substances can also impair functioning and dysregulate behavior, threatening others’ safety and well-being, for example, while driving or caring for children. Also, the combined use of substances (e.g., using alcohol or sleep aids while taking pain medications) or prescribed medications with ingredients that could interact with substances (e.g., Scott et al., 2020; Weiner et al., 2022) can be life-threatening or debilitating, therefore impairing the ability to work, care for family, and function overall (Hasin et al., 2017; O’Brien et al., 2021; Sarvet et al., 2018; Westling et al., 2022). As such, the widespread use of substances associated with negative health, public safety, developmental, and economic consequences require a coordinated response by multiple stakeholders in this arena, therefore, elevating substance use to a national public health level (Substance Abuse and Mental Health Services Administration and Office of the Surgeon General, 2018).

**Fig. 1:**
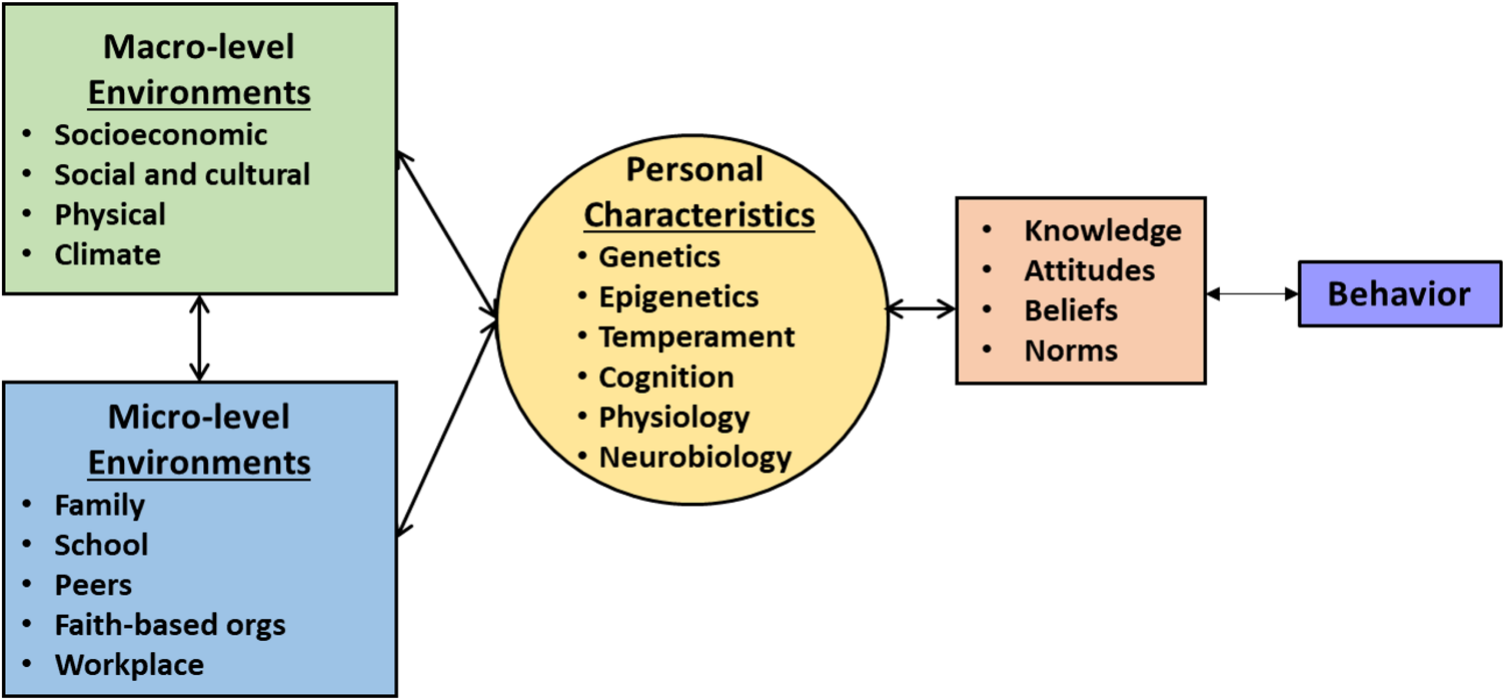
Etiological Model

A public health framework positions substances, along with any adulterants, as ‘etiologic agents’ in that they impact the functioning of the brain and can impair functioning of other vital organs and overall health through their direct effects. The host includes children, adolescents, and adults of all ages, particularly those vulnerable as a function of their own neurobiological makeup and their micro- and macro-level environments (see Fig. 1). Understanding the framework and its components can guide development of interventions that prevent not only initiation of substance use, but also to attenuate the consequences of use via treatment, needle exchange programs, administration of Narcan, or recovery supports. The literature is replete with reviews and recommendations for these more tertiary, enforcement, regulatory, and harm reduction approaches. Here, we focus our policy recommendations strictly on primary prevention—to avert developmental trajectories away from substance use in the first place—to address the vulnerability and resilience factors that affect those pathways and have potential to be manipulated with evidence-based interventions and policies. The following section delves further into the etiological model of SUD/OUD to identify opportunities for early intervention and subsequently emphasize the need for a national strategy to support their implementation, sustainment, and scale-up.

## What Science Tells Us About Pathways to Substance Use and Substance Use Disorders

Scientists have been working to identify the personal and environmental conditions that are conducive to substance use, and to understand the nature of “resistance” factors that protect individuals from initiation or later escalation of use (Rose et al., 2019; Sloboda, 2015; Vanyukov et al., 2016). Two NIH institutes – the National Institute on Drug Abuse and the National Institute on Alcohol Abuse and Alcoholism – have extensively supported studies of the etiology of substance use, as well as abuse and dependence. The research funded by these two agencies alone have produced abundant knowledge on the problem and its precipitants, which have led to significant advances in its prevention and treatment (e.g., Kiluk & Carroll, 2013; NIAAA, 2020; NIDA, 2021).

Development of substance/opioid use disorders (SUD/OUD) is commonly preceded by a variety of psychological and behavioral problems, including academic failure, conduct problems, sensation-seeking, impulsivity, anxiety, depression, and stress-related disorders (United Nations Office on Drugs and Crime, 2018). These problems often arise due to detrimental social determinants of health (e.g., poverty, family dysfunction, inequities, structural racism, lack of community supports) that create environments unable to foster effective cognitive, coping, and prosocial skills in young people. These environments tend to be rife with opportunities and influences to engage in problematic behaviors; for example, there may be a lack of supervision, deviant peer groups, marketing of abusable substances, community violence. The risks have a universal impact but are infinitely more impactful for individuals with a history of adverse childhood experiences (ACEs) (Fishbein & Ridenour, 2014). The ACE Study (Felitti et al., 2019) reported that individuals who experienced 4 or more ACEs—12.5% of the population—were 1030% more likely to partake in intravenous drug use. Further research found that the ACE-related population attributable risk for overdose deaths from heroin and synthetic opiates was 78%. Caution is warranted in the interpretation of these results because the findings were based on retrospective data; however, they call attention to the need for further study and subsequent action.

The presence of protective factors, on the other hand, can mitigate vulnerability in the face of adversity. Examples of protective factors include warm and involved caregivers, academic competence, neighborhood and school attachment, strong self-regulatory and social competency skills, effective anti-drug and harm reduction policies, job opportunities, and more (Hill et al., 2010). An understanding of this sequencing has led to the development of numerous prevention strategies designed to support healthy parenting, strengthen cognitive controls, foster prosocial behaviors, teach adaptive ways of managing stress, provide opportunities for mobility, reduce health and educational disparities, and promote trauma-informed practices in communities and child-serving institutions (e.g., LoBraico et al., 2019; Sanders et al., 2017). Programs and policies of these sorts require implementation across the life-course with special emphasis during key developmental transitions (e.g., early childhood, and adolescence) to provide for a safe, nurturing environment for healthy development.

**Fig. 2:**
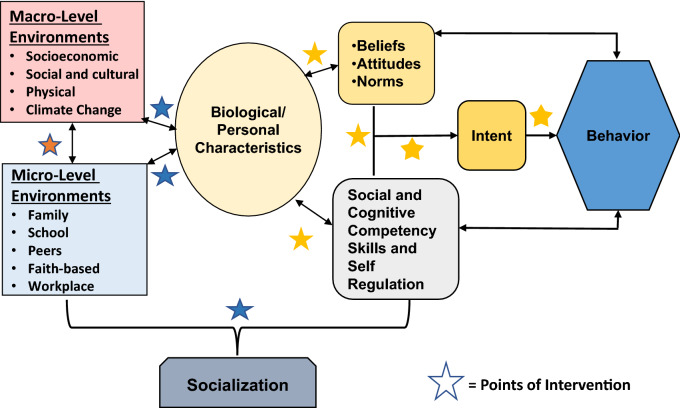
Opportunities for Intervention

A review of the research literature suggests an etiology model that is based on socialization processes, their impact on and interaction with personal characteristics, and other factors that either foster or hinder optimum development, wellness, and acquisition of healthy life skills. Figure 1 illustrates this process, demonstrating the bidirectional nature of the socialization process whereby the biological and emotional characteristics of individuals interact with their micro- and macro-level environments to influence attitudes, beliefs, norms, and behaviors. Furthermore, there is a bidirectional interaction between these two environments suggesting they can exert both positive and negative influences (Sloboda, 2015). Thus, extreme poverty, social upheaval, racism, and even climate change can negatively impact the micro-level environments, thus creating stressful situations and interactions between individuals and families. Figure 2 further demonstrates opportunities for interventions to mediate these stressors and enhance positive socialization. Taking this knowledge into account will enable the formulation of policy solutions that are appropriately targeted to different subgroups and environmental contexts.

## An Evidence-Based National Policy Response

In the course of rapidly responding to the devastation of SUD and the unprecedented rise in related deaths and family disruptions, the nation has not invested in preventive strategies as heavily as the epidemiology and prevention science dictates. Evidence-based interventions have been developed to target proximal factors that increase risk for SUD (e.g., behavioral problems, trauma, lack of parental involvement). And at the population level, a variety of broader approaches are available, including enforcement of policies related to access and availability of tobacco, alcohol, cannabis, and other harmful substances, as well as policies that affect prevailing conditions that particularly underlie the SUD crisis (e.g., child poverty, family economic instability, systemic racism).

Research has convincingly established that these multiple life-course conditions, influential in whether an individual will initiate the use of substances or develop a SUD, are alterable and, in many cases, preventable. Protective conditions and resiliency can be strengthened, while detrimental influences can be attenuated or eliminated altogether. Recognition of these facts by policymakers and the public will direct us to more effective policy solutions and could lead to wiser capital expenditures, all with the potential to make a measurable dent in the problem (Eisenberg & Neighbors, 2007). In addition to providing a nurturing environment where children can thrive and avoid substance use and other negative behaviors, employing proactive strategies when problems arise in children and adolescents include screening, early identification of the warning signs, and referral to needed services (NIDA, 2020). Addressing both the prevailing and proximal conditions that often give rise to behavioral problems early in life, prior to entrenchment of SUDs has potential to significantly reduce SUD and shrink the OUD crisis, as well as prevent transmission to subsequent generations (Neppl et al., 2020).

The 2022 National Drug Control Strategy discusses prevention under “Prevention and Early Intervention,” stating, “ensuring that school-aged children have access to universal prevention programs designed to prevent use before it starts, prevention services that focus on children at higher risk for use or those that have started using drugs, and when necessary provide referral to treatment and recovery support is essential to support the health, well-being, and futures of the Nation’s 74 million children” (p.21). Three principles are laid out for addressing prevention: Preventing Substance Use Among School-Aged Children is Effective; Preventing Substance Use Among Young Adults Promotes Overall Health and Preventing Youth Substance Use Requires Community Level Interventions. Under each principle are several general objectives (pp. 21–29) however, overall, the Strategy lacks details as to how to implement these principles and their objectives.

Accordingly, this paper builds on the Strategy by emphasizing the importance of: (a) preventing conditions that pose a significant risk for SUDs and their associated social and health consequences; and (b) sustained, systematic and high quality delivery of evidence-informed programs and public health policies. The nation has capacity to jump-start a path to better child and family well-being, thereby averting trajectories away from substance use and eventual SUD/OUD and other behaviors that impact the lives of those affected and their families. Presented below is a plan that incorporates eight significant and interrelated components and corresponding policy recommendations.

### Broad and Systematic Investment in Evidence-Based Programs

The three most important environments affecting young people’s development are families, schools, and neighborhoods (NASEM, 2019). Building a comprehensive prevention system requires implementation of evidence-based programs and policies in each of these domains to mitigate harmful influences, including investing in research and development of new promising practices. This integrative approach brings together all societal sectors working on some aspect of prevention and/or public health with stakeholders and community leadership to develop a plan that addresses a wide range of risk factors. The return on investment will be improvements in multiple child outcomes, from fewer problems in early grade school to reductions in juvenile justice involvement that otherwise drive pathways to OUD as well as the harms associated with the use of other substances. Benefits will also be seen in financial returns in terms of reduced cost and burdens associated with health, mental health, child welfare, juvenile justice, and social service systems (Eisenberg & Neighbors, 2007). A comprehensive prevention system has the potential to mitigate the impact of “toxic” social conditions on families and community institutions, such as excessively harsh school disciplinary practices, that ultimately give rise to behavioral problems such as SUD, delinquency, and other concerns.

Conditions that underlie these issues can be comprehensively and effectively addressed by optimizing and scaling evidence-based family, school, and community level preventive interventions and policies designed to reach a range of populations. Approximately 20 evidence-based family-based programs have been shown to significantly improve the quality of family life to prevent many behavioral problems, including substance misuse, antisocial behavior, anxiety, depression, risky sexual behavior, school absences, and academic performance (Leslie et al., 2016). Numerous tested and effective school-based interventions can prevent these problems as well, from early childhood into adolescence (Hawkins et al., 2015; NCR/IOM 2009 Report) by improving school climate and school bonding. Also, more than 40 policies have proven benefits in increasing families’ economic and social stability (Spencer & Komro, 2017) that, in turn, reduce substance use. Some programs shown to specifically reduce behavioral problems and substance use include The Incredible Years (Leijten et al., 2018; Overbeck et al., 2020), the Good Behavior Game (Troncoso & Humphrey, 2021), Triple P–Positive Parenting Program (Li et al., 2021), Nurse-Family Relationship (Kitzman et al., 2019; Miller, 2015); the Botvin LifeSkills Training (LST) program (Valasco et al., 2017), Project Towards No Drug abuse (TND) (Sussman et al., 2014), and Multisystemic Therapy (van der Stouwe et al., 2014). Extensive analyses of the costs and benefits of these programs indicate that most save far more than they cost in reduced healthcare, criminal justice, mental health, and educational costs, and in increased income among recipients (Washington State Institute for Public Policy, 2016). An integrative approach deploying a blend of programs at all levels improves effectiveness and reach.

In addition to interventions targeted toward individuals and families, community-based strategies have been shown to reduce SUD and improve overall health outcomes. For the most part, child-serving agencies such as child welfare, education, and health care are not currently well-coordinated, do not share data, and function more or less independently. Community-based approaches that address these gaps support coordinating systems of care across different agencies and providers, invoking prevention at a system-wide level. One such system, the Community HUB model (Agency for Healthcare Research & Quality, 2021; Zeigler et al., 2016), involves identifying an at-risk population (e.g., families impacted by caregiver SUD, poverty, or marginalization) and encourages agencies to identify and refer all eligible families to a single agency that provides a home or community visit by a case manager who assesses family needs and facilitates appropriate referrals with an emphasis on evidence-based service. A Community HUB, for example, might train their case managers in parenting support services and engage families in evidence-based parenting support services.

Another exemplary community-wide strategy is Promoting School-Community-University Partnerships to Enhance Resiliency or PROSPER (Spoth et al., 2017), also referenced in the Strategy (p. 20). PROSPER is based on a multi-tiered structure consisting of (a) community teams, (b) a state-level management team, (c) a prevention coordinating team, and (d) a national-level tier, the PROSPER Network Team. The Network Team includes prevention scientists, faculty, and professionals involved in the development and original implementation of PROSPER in Iowa and Pennsylvania. PROSPER combines family- and school-based prevention approaches and targets families with middle-school children. Community teams select a family and school program from a menu of PROSPER-supported, evidence-based programs (EBPs) and manage program delivery. PROSPER has been shown to reduce delinquency and substance use during high school and promote family management practices and parent–child affective quality.

Finally, an evidence-based system of services that is most widely cited, including in the Strategy (p.20), is Communities That Care (CTC; Kuklinski et al, 2021). CTC is designed to reduce levels of adolescent delinquency via the selection and use of evidence-based prevention programs tailored to a community’s specific risk and protection profile. Through training events and community activities, CTC aims to produce community level changes in the service system characteristics, including increased collaboration among providers and greater adoption of evidence-based programs that address risk and protective factors the community prioritizes. In turn, reductions in community risk factors reduce adolescent delinquent behaviors.

Sufficient and sustainable investments in the implementation, broad availability, and ongoing evaluation of these interventions and health-level policies are needed to increase capacity of government agencies, practitioners/clinicians, schools, and communities. Offering a menu of evidence-based programs, implementation guidance, evaluation services, and continuous learning opportunities can be supported by well-coordinated efforts across systems (Fagan et al, 2019). Multiple layers of influence are the focus of policies that are articulated in real-world terms, define governance and support systems, outline and resource delivery mechanisms, and ensure feedback loops between governance-support-delivery systems for optimal implementation and scaling. Each layer must work interactively to create a hospitable environment for best results. This process is known as the Interactive Systems Framework for Dissemination and Implementation (Wandersman et al., 2008), a framework that has been adopted by the CDC (The Centers for Disease Control and Prevention, Division of Violence Prevention). Specific features supportive of systems change are delineated below.

*Establish a Sustainable Funding Stream to Support Prevention* Programs exist within networks of social ecological systems in which people are embedded. To be successful, even the most effective off-the shelf programs require systems-level buy-in and support. Administrations involved (Office of National Drug Control Policy (ONDCP), Centers for Disease Control and Prevention (CDC), Substance Abuse and Mental Health Services Administration (SAMHSA), National Institutes of Health (NIH), Human Resources and Services Administration (HRSA)), and possibly the Centers for Medicare & Medicaid Services under the Department of Health and Human Services, may use their budget authority to encourage its agencies to allocate funds towards prevention through a lead federal entity. This entity could convene agencies around this Call to Action to provide training and technical assistance grants to support high quality implementation and evaluation. It may also convene stakeholders who are vital to include in these discussions and that can rally administrations to focus on prevention.

*Capitalize on Existing Infrastructure *Collaborating with the single state alcohol and drug agency in each locality will provide communities with the resources and tools needed to build supportive infrastructure for customizing and implementing prevention programs. Each Single State Agency (SSA) has a member in the National Prevention Network (NPN) and would also work in coordination with the education, public health, and other relevant systems at the state and local level. This feature avails itself of existing resources and directs communities and agencies toward the development or expansion of state-supported and community-based prevention infrastructure.

*Focus on Evidence-Based Investments with Continuous Quality Improvement Implementation* As evidence-based programs are rolled out, ongoing performance accountability is required (e.g., assessment, feedback, and technical support). In general, the larger and longer term the investments (such as an endowment fund for prevention) the better in terms of population level impacts. Investments in specific programs may also be useful but only temporarily, depending on trends in substance use patterns and underlying conditions.

*Establish a Screening and Referral Infrastructure in Health and Social Service Agencies and Educational Institutions* Educate and equip a range of professionals working with youth and families considered at-risk and who reside in communities with a high prevalence of deleterious conditions. This workforce spans educational, justice, public health, primary care, child welfare, and other sectors/systems. Each contains its own infrastructure; however, most have not accommodated the knowledge and practices generated by prevention science, and they rarely collaborate to provide a concerted and consistent response to individuals and families in need. To fill this gap, each sector can be equipped with well-tested tools for conducting health risk appraisals and preventive counseling throughout development (Matson et al., 2021).

*Embed Strong Intermediary Support* Embedding intermediary organizations is vital for the assessment of and community-driven response to local needs. Such organizations offer a menu of programs and policies that specifically address the needs of any given community, and they provide ongoing consultation and technical assistance on selecting, implementing, and evaluating prevention efforts. They are typically able to stay well-connected to emerging evidence, have a strong focus on equity and community inclusion, and be able to work at the state-level to guide ongoing system design and improvement. The EPISCenter (Pennsylvania State University, 2020) and Impact Center in the University of North Carolina’s Frank Porter Graham Child Development Institute exemplify how investments in education, training, and technical assistance mechanisms impact communities that stand to benefit from the translation, implementation, and evaluation of evidence-based strategies.

### Public Health Policies

Public health policies available at the community-level can support and upgrade health care, education, housing, employment, and social nets to enable full participation of all community members in the economic, social, and physical health of the population. Enhancing public awareness of the social determinants of health and their relationship to risk for developing SUD/OUD is a key feature of a public health strategy. Other strategies that have shown efficacy include legal age requirements for use of alcohol and now cannabis, as well as stricter prescription opioid prescribing regulations and more concerted enforcement of those policies. Service delivery systems are particularly needed to systematically address societal factors that often lead to inequities in health and social environments, placing individuals and families at disproportionate risk for negative outcomes (see the Brandeis Opioid Resource Connector). Drug Free Communities grants exemplify a federal policy with widespread impact through the provision of services at the local levels (https://obamawhitehouse.archives.gov/ondcp/Drug-Free-Communities-Support-Program). And collection and systematic evaluation of data reflective of the trends and their geographical and demographic distribution is another aspect of public health that can help to more precisely target the underlying precipitants of inequitable conditions that can lead to SUD/OUD. The National Survey of Drug Use and Health (NSDUH) and Monitoring The Future (MTF) are two databases that have enhanced our understanding of prevalence rates, areas of concentration, and prevailing risk and protective conditions in those regions of the country, serving to guide preventative measures. Additional investments in such data systems are needed to capture more meaningful information and to increase the utility of that information. Public health departments at the state and local levels can be equipped with these capacities with the appropriate policy changes, with the promise of exerting population level benefits.

### Invest in Further Prevention Research to Address Outstanding Questions

Many preventive interventions have demonstrated the potential to disrupt pathways to SUD and OUD. Nonetheless, there are inconsistent results and many outstanding questions. For example, not all recipients respond well to the tested interventions, which means that more effective intervention models, targeting strategies, and implementation processes are needed. There is also a need for translational research to determine how to move the science more rapidly from program development to efficacy testing to routinized delivery systems. In addition, when prevention programs are implemented across diverse settings and contexts, oftentimes original outcomes are not replicated (e.g., Overbeck et al., 2020; Foxcroft et al., 2017); thus, guidelines for rigorous and transferable methodologies as well as monitoring implementation processes are sorely needed.

A vitally important consideration is that risk factors are more prevalent in disadvantaged and marginalized communities due to broad social and structural influences on health outcomes, including poverty and discrimination (Dwyer-Lindgren et al., 2017). Research is needed to develop and experimentally evaluate comprehensive interventions designed to reduce health disparities. At the same time, experimental research is needed to evaluate systems change and policy strategies for reducing poverty, inequities, and discrimination that, in large part, are the primary determinants of poor health outcomes including OUD. Furthermore, research is needed to better understand the processes and mechanisms underlying resistance or protective factors found to be associated with maintaining behavioral health and with the ability to avoid SUD/OUD in the face of adversity. And importantly, for all aspects of the research – from the formulation of research questions and hypotheses to the conduct of the study protocol and interpretation and translation of findings – it is imperative that the community drive the process as trusted partners. Otherwise, we risk further perpetuating the inequities, disparities, and injustices that we seek to eradicate. To accomplish this goal, a high priority is the conduct of research that elucidates how best to engage members of the community, particularly those that may be the most difficult to reach. (See https://www.npscoalition.org/consortium-to-advance-prevention-solutions-to-the-opioid-crisis-capsoc for a letter outlining prevention research needs).

To advance these lines of inquiry, an advisory group can be established by NIDA and/or other federal funding agencies (e.g., SAMHSA, CDC, HRSA) to identify gaps in knowledge regarding the development, implementation, evaluation, and dissemination of an array of preventive interventions and policies to address outstanding scientific questions, with direct implications for more efficient and effective uptake in communities. The Consortium to Advance Prevention Solutions to the Opioid Crisis (https://www.npscoalition.org/consortium-to-advance-prevention-solutions-to-the-opioid-crisis-capsoc), a large national group of senior SUD experts, can be tapped by federal agencies to guide this effort. Increased federal funding of studies focusing on programmatic methods is also needed to utilize current and emerging knowledge on pathways to OUD to quell the opioid crisis and other substance use issues (Bipartisan Policy Center, 2019). There are myriad ways to implement this protocol either at the department or agency level or through contracting during disbursement of funds to states/localities. Doing so will facilitate evaluation of how the funds are being used and whether objectives are achieved.

### Invest in Training of Prevention Professionals

The gap between prevention science and practice in the field of substance use prevention has begun to narrow over the past two decades. California’s Substance Abuse Prevention Workforce Development Survey Report 2013 (Center for Applied Research Solutions, 2013) recommendations exemplify this trend, including to: (1) create professional and/or educational avenues for individuals to pursue substance use prevention as a viable, credible, and transferrable career; (2) enhance the opportunities and systems to build the capacity of the substance use prevention field; and (3) promote and foster leadership for substance use prevention.

Clearly, the results of research regarding effective strategies to address substance misuse and other high-risk behaviors must be made available to the practice community for this work to exert an effect. Existing resources include the first National Conference on Drug Abuse Research: Putting Research to Work for Communities and the associated guide, Preventing Drug Use Among Children and Adolescents (Sloboda & David, 1997) sponsored by NIDA; the creation of registries of effective programs such as Blueprints; the support of community coalitions such as Drug Free Communities; the publication of the International Standards for Drug Use Prevention by the United Nations Office on Drugs and Crime (2013); and the development of the Universal Prevention Curriculum by Applied Prevention Science International with funding from the U.S. Department of State. Furthermore, in 2018, SAMHSA established the Prevention Technology Transfer Centers (PTTCs) “to improve implementation and delivery of effective substance misuse prevention interventions and provide training and technical assistance services to the substance use prevention field” (the PTTC Network). Another resource provided by the High Intensity Drug Trafficking Area (HIDTA, an ONDCP initiative) is A Division for Advancing Prevention and Treatment (ADAPT) offers expertise, trainings, and technical assistance to translate, implement, and evaluate substance use prevention strategies within each unique community. And the International Certification and Reciprocity Consortium (IC&RC) provides credentialling to prevention professionals from around the world for receiving a passing grade on an examination to gauge knowledge and competencies with 46 States participating in the certification program. And yet another source for credentialling is the National Prevention Science Coalition to Improve Lives (NPSC), certified by the American Psychological Association (APA), to provide trainings and courses to a broad audience, including prevention practitioners and clinicians to further professionalize the field and incentivize practitioners and scientists to engage more deeply in the science advocacy and policy process. Preparing the prevention workforce with formal training in prevention science and its application to practice must be systematized (Coyne et al., 2008; Eddy et al., 2005; Miovsky et al., 2019).

Steps toward developing a viable prevention workforce include the following: (a) Forming an advisory group (e.g., by the National Academy of Sciences, Engineering, and Medicine) to identify and review existing prevention science training programs in colleges and universities and those offered by prevention education/training organizations, as well as materials such as those from IC&RC and SAMHSA that provide listings of competencies of prevention professionals (Epstein & Hundert, 2002); (b) Providing support for the development of instruments to be used by the PTTC to conduct a training needs assessment survey of prevention professionals in every state to determine gaps in knowledge between the science and its application to prevention practices and competencies; (c) Increasing federal funding for states to incentivize the provision of continuing training of prevention professionals and for university students who wish to major in prevention science tracking to either research or practice; and (d) Working with the Department of Labor to develop a job classification for prevention professionals.

The following websites offer high quality prevention practitioner trainings:

- Prevention Technology Transfer Center Network (PTTC): https://pttcnetwork.org/
- Evidence-based Prevention and Intervention Support (EPIS) Center: https://epis.psu.edu/
- Applied Prevention Science International: https://www.apsintl.org/
- A Division for Advancing Prevention and Treatment (ADAPT): https://www.hidta.org/adapt/
- Coalition for the Promotion of Behavioral Health (CPBH): https://www.coalitionforbehavioralhealth.org/
- Community Anti-Drug Coalitions of America (CADCA): https://www.cadca.org

### Enhancing State and National Epidemiologic Monitoring and Surveillance Systems

In 1991, SAMHSA assumed oversight of several important monitoring systems including the National Household Survey on Drug Use and Health (NHSDUH), the National Substance Use and Mental Health Services Survey (NSUMHSS), the Treatment Episode Data Set (TEDS), and a national surveillance system, Drug Abuse Warning Network (DAWN) (SAMHSA, 2022). The National Institute on Drug Abuse (NIDA) supports the Monitoring the Future Study and the National Drug Early Warning System (NDEWS). These data sets, among others (such as those for wastewater analysis and overdose spike warning and response systems) have the potential to inform, not only the service needs of the population, but also to assess accessibility and barriers to the utilization of services (for more information see the European Monitoring Centre for Drugs and Drug Addiction webpage on “key indicators” (https://www.emcdda.europa.eu/topics/key-indicators_en)). Furthermore, the data serve to monitor the utilization and short-term outcomes of the services and can help identify service insufficiencies and gaps. A surveillance system that would include existing systems such as DAWN and the National Drug Early Warning System would provide timely alerts of emerging patterns of substance use including new substances, new ways to administer existing substances and new users of these substances. The latter system also has potential to analyze seized drugs to determine their potency, contents, and added adulterants that have health implications (Browne et al., 2021).

**Fig. 3:**
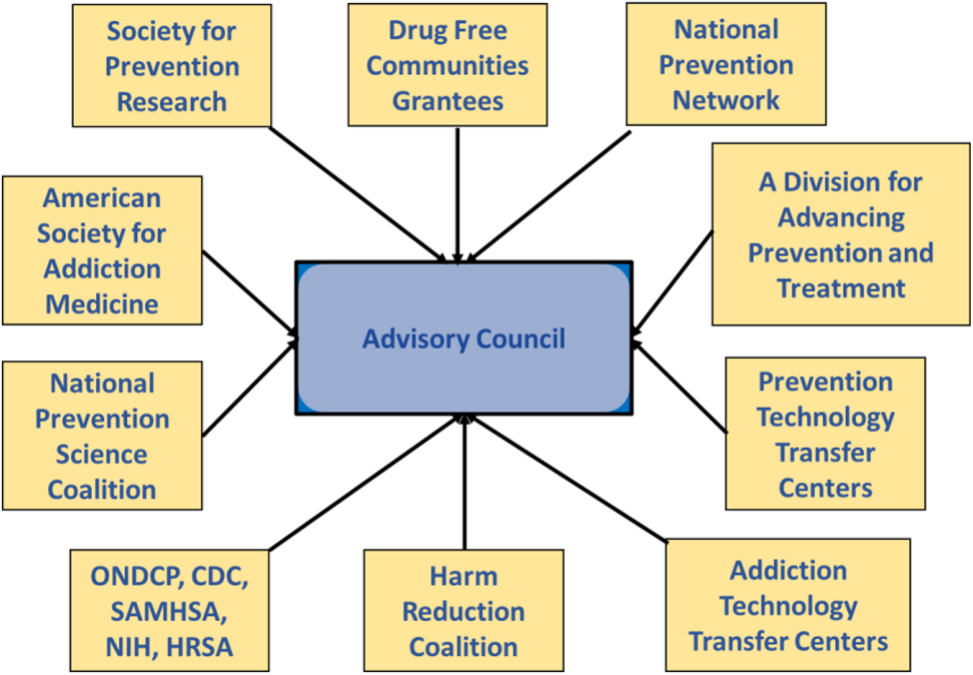
Interagency-Institution Coordination. Advisory Council Engaging Key Organizations. A deep understanding of research and evidence-based practices and policies increases the potential to positively impact those affected by the opioid crisis, stemming overdoses, and ensuring preparedness for future drug-related issues. It is especially essential that federal, state, and local leaders critically examine current approaches for addressing opioid addiction and overdoses to ensure the policies and programs implemented have maximal impact

Establishing these systems requires creation of an advisory group comprised of epidemiologists, prevention, and treatment professionals and staff of NIDA, NIAAA, NIMH, CDC, ONDCP, and SAMHSA to review existing monitoring and surveillance systems sampling procedures, data collection instruments, reporting formats, and so forth, to make recommendations on improving these national systems (Fig. 3). They should include the existing systems mentioned above, along with others that would guide and assess prevention programming. This advisory group should be expanded to include representatives of state-level prevention, harm reduction, and treatment organizations (e.g., National Association of State Alcohol and Drug Abuse Directors, National Prevention Network) and representatives from federally and State-funded community coalitions (e.g., Drug Free America, Communities That Care, PROSPER) to review the advisory group recommendations as they pertain to the needs of states and communities.

Additional recommendations include the development of ongoing reporting formats to determine “need” and “demand” for prevention, harm reduction, and treatment services at the community level. A review group can be established to determine the degree to which the needs are addressed in communities across the country and formulate recommendations for studies that shed light on their origins. Providing funding for these recommended studies can lead to solutions to problems that arise and will reduce the gaps that are identified. With the above-mentioned advisory group and including the Drug Enforcement Administration, CDC, and the HIDTA, establishing a ‘street to lab’ surveillance system will facilitate monitoring of the content of substances seized on the street and to harmonize data collected, such as geocoding. And finally, creating a public communications system will inform local communities, prevention, harm reduction, treatment services providers, departments of public health, health care providers, and medical examiners/coroners about the content of seized substances in their areas and steps that can be taken to prevent their proliferation.

### Enhance the Usability and Reach of National Preventive Intervention Registries

There are several existing registries populated by hundreds of programs, interventions and policies that have been subjected to evaluation and rated for their effectiveness in reducing substance use and related problems (e.g., Blueprints for Healthy Youth Development (University of Colorado), What Works in Social Policy (Social Programs That Work, 2022), Results First Clearinghouse (The Pennsylvania State University, Results First Clearinghouse Database, 2021), Crime Solutions (National Institute of Justice), Home Visiting Evidence of Effectiveness (U.S. Department of Health and Human Services), Title IV-E Prevention Services Clearinghouse (U.S. Department of Health and Human Services), and SAMHSA's Evidence-Based Practices Resource Center (U.S. Department of Health and Human Services). They are not well utilized, however, due to their complexity and lack of intensive guidance for the user to successfully implement the programs. There is also insufficient reach to communities and policymakers who stand to benefit from the information they provide.

A national clearinghouse that broadly addresses the priorities stated by the Evidence-Based Policymaking Act is needed to provide infrastructure and detailed implementation guidance for rigorously evaluated programs and policies shown to reduce substance use (NPSC, 2021). To date, the nation has invested in many drug control strategies that either have not been evaluated or have not produced sufficient effect sizes to justify their implementation or continuation. The same is true for programs to reduce mental health problems. Such a clearinghouse could direct policymakers and community stakeholders toward programs shown to work to avoid waste of precious dollars and resources. The idea behind such a clearinghouse is to organize the large reserve of data on evidence-based programs and policies populated by other existing registries and databases within a platform accessible to a range of end-users (e.g., community stakeholders, practitioners, policymakers, governmental agencies) working toward a solution to the exorbitant rates of opioid and other substance use. The clearinghouse would provide detailed and customizable guidance to walk end-users through the implementation process and ensure program selection aligns with community needs. There are several logical homes for this Clearinghouse, such as the Office of Management and Budget (OMB), CDC, ONDCP, or SAMHSA, providing cross-agency access to such a well-organized and inherently usable data infrastructure for evidence-based programs and policies. See https://www.npscoalition.org/ebp-clearinghouse-proposal for further information.

### Building a Strong Community-Based Infrastructure to Support Preventive Interventions

To effectively address SUD and OUD at all levels, a multi-phased plan is needed to develop and implement a national community-based integrated system of evidence-based substance use prevention services by the year 2030. Five initial phases (a-e below) are recommended to begin the process of making systemic changes to how to approach substance use and its consequences.

- Identifying or creating a model community prevention, early intervention, and treatment infrastructure is critical. Communities will benefit from advanced training in prevention science and its application to practice as well as in creating and supporting community partnerships to address prevention within the social, political, and cultural framework of their communities. This activity would inform the development of a model representing the continuum of services that are recommended and the concept of supporting three or more demonstration sites to be monitored, evaluated, and refined.
- Developing a ‘standard’ problem assessment process and delineating components of a strategic planning process can be accomplished by convening a two-day meeting of approximately 25 leaders, including substance use epidemiologists, prevention, and treatment researchers and practitioners, law enforcement, judiciary, schools, families, communities, policy makers, and health services. The purposes of such a convening are to refine the model of a community-based integrated comprehensive substance use service delivery system, develop a summary of challenges and potential solutions, and draft an initial framework for a strategic plan to develop and evaluate demonstration systems. The plan will outline recommendations and implementation steps, and funding sources. Public health policies and prevention services can only be effective when encompassed within the framework of a comprehensive plan. As such, this approach will emphasize the utility of prevention as a vital component of the overarching plan, from primary prevention and harm reduction, to treatment, and recovery.
- Expanded stakeholder involvement is a required element of a comprehensive strategy, integrally incorporating community input and then disseminated to the public. A team would seek support for a community-based comprehensive service delivery system and for the prospect of demonstrations. These additional planners will likely represent various public sectors (federal, state and local governments) and would help to forge implementation approaches.
- As detailed in #4 above, training and technical assistance protocols are needed to professionalize the prevention workforce and assist communities with building an implementation system to support prevention, including health and social services, schools, parent groups, businesses, law enforcement and the judicial system.
- A community level assessment systems, as part of a comprehensive prevention system, requires screening, early intervention, referral, and monitoring systems that link service needs with research-based programming.

Ultimately, systems change and the utilization of tools, trainings, and assessment protocols to support that change must be community-driven. Scaffolding in the form of policy reforms, technical assistance, education, and funding will be needed from governing and administrative bodies at all levels to systematize, sustain and scale these efforts. The EPIS Center at The Pennsylvania State University (http://epis.psu.edu) is one case in point.

### Building a Community Infrastructure to Achieve Successful Outcomes

**Fig. 4:**
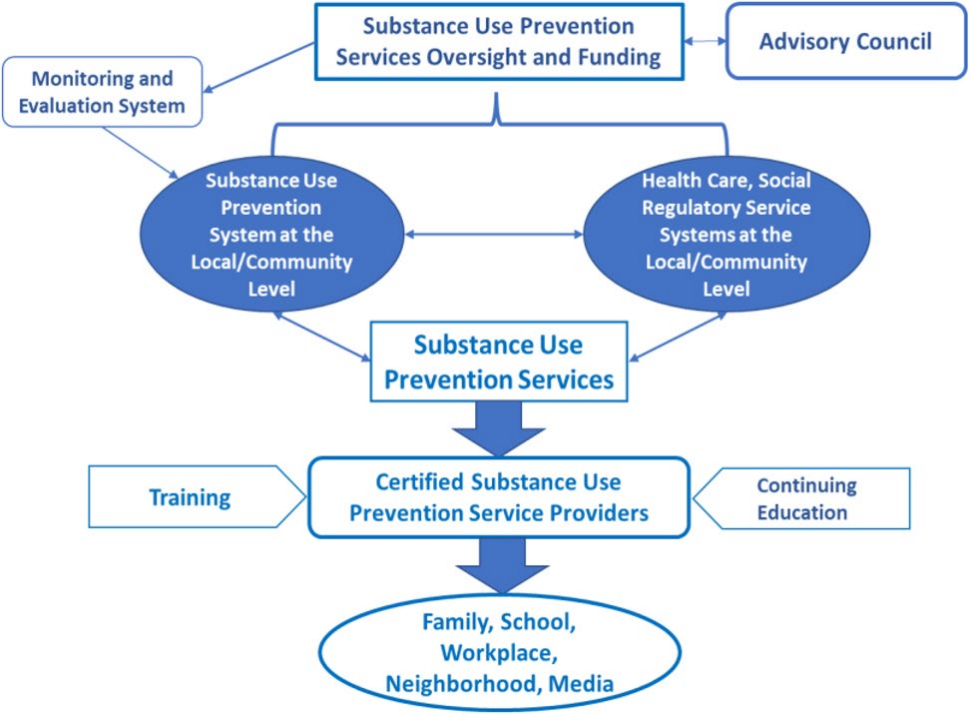
Proposed Structure for Model Substance Prevention Service Delivery System

A strong community-based infrastructure, such as those used in CTC and PROSPER, is needed to support delivery of preventive interventions via both public and private investments. A 5-phase process is presented that would feed into the ***Substance Prevention Service Delivery System*** (see Fig. 4) described below for the effective and cost-efficient implementation of interventions known to reduce risk for SUD/OUD. Engagement of administrative entities, such as departments of health and human services, departments of public health, governor’s offices, and local agencies to facilitate and scaffold infrastructure at the community level is necessary for such a system to be effective. The phases include:

- Families, community members and professionals across sectors convene to decide on goals, programs, desired outcomes, and actions for successful implementation efforts. Sectors may include health care, law enforcement, schools, and the judiciary among others.
- Their ideas and plans are shared with the public and local officials for additional input and support for the community-based delivery system that best reflects the needs and preferences of the community.
- Service providers such as medical offices, mental health and family support services, and school counselors are trained in how to best provide these prevention programs and services.
- A tracking and assessment system for screening, early intervention, referrals, and monitoring is set up for relevant agencies and other settings (such as family practice or pediatric offices, schools, and family courts) to link teens and families with evidence-based practices.
- A document is produced that provides a set of instructions to guide the establishment, monitoring, evaluation, and improvement of the prevention services delivery system to support parents and their children.

There is an argument to be made for the necessity of cost–benefit analyses to support the economic viability of this approach, by demonstrating the significant cost savings of preventing problems such as SUD prior to their entrenchment (Crowley et al., 2018). Healthcare, child welfare, mental health, educational, public safety, and other systems are significantly burdened when investments are not made in primary prevention approaches, leading to wasteful spending (Eisenberg & Neighbors, 2007). Supportive infrastructures work to avert trajectories away from negative outcomes and, in effect, promises to substantially reduce costs associated with these burdens. To motivate or incentivize investments in this undertaking, it is important that this body of evidence reach critical targets; e.g., governors, state and local administrators, and community stakeholders.

## Building a National Comprehensive, Integrated Substance Use Delivery System to Scale and Sustain Evidence-Based Programming

Prevention science offers an evidence-based approach to tackle the underlying causal/structural/systemic conditions that contribute to the problems we wish to prevent, including SUDs. In practice, all human services systems are nearly entirely focused on tertiary, not primary, prevention. And even when prevention-minded solutions are considered, insufficient attention is paid to access, availability, efficacy, and motivation to deliver and receive such programs. Many people do not seek preventative care due to stigma or inaccessibility. Moreover, identifying people at-risk and offering prevention services has not been very useful, in large part because few are available and motivation to seek preventive care is lacking. We need to expand the range of interventions to develop, evaluate, and, most importantly, implement effectively and equitably. Advances in prevention science have much to offer; they have led to the development of numerous effective preventive interventions, as well as guidance regarding how to apply that knowledge to actionable prevention and promotion frameworks. And yet, those advancements are not the only battle to be fought because we still need to ensure availability, access, efficacy, and motivation. It is time to increase the prevalence of these services (scaling) and increase awareness of their benefits and utility (dissemination and policy translation). On the other hand, service expansion alone will not fix the problems. The whole system of human services needs to be transformed. Accordingly, we propose a national comprehensive preventive service delivery system.

One framework from Fagan et al. (2019) presents a strategy for embedding and scaling up evidence-based preventive interventions across public human service systems by implementing a common set of supportive competencies. This model helps us to understand how the systems’ contexts and capacities impact the degree to which a continuum of prevention solutions can take hold. To accomplish these objectives, they state it is necessary to build: (1) developer and funder capacity; (2) public awareness; (3) community engagement and capacity to implement them at scale; (4) public systems leadership that supports primary prevention; (5) a skilled workforce capable of delivering the programs; and (6) data monitoring and evaluation capacity. In the foregoing analysis, we have built from this model an elaborated set of recommendations specifically directed toward solutions to the SUD crisis and offer specific actions and players required for executing them.

Critically, legislation is needed to enable societal sectors and systems to be more responsive to the needs of the U.S. population by supporting a national system of services, from universal prevention programming to more targeted and indicated interventions. Supports should be designed to reach different groups; e.g., nonusers (to reinforce their non-use), those vulnerable to initiation or who have already initiated (to prevent progression to abuse), and those with a SUD who opt not to enter treatment or are receiving treatment and require reintegration into the community.

Funding for substance use prevention services currently derives from a variety of sources but primarily from federal, state and local governments. At the federal level, the major funding sources are grants from SAMHSA and the CDC, while data to inform the need for prevention services are generated by these two agencies plus the National Institutes on Drug Abuse (NIDA) and Alcohol and Alcoholism (NIAAA) as well as the DEA. State and local funding comes from agencies that are directly related to substance use or to public health or mental health. In addition, information regarding prevention—whether it pertains to research as to the effectiveness of prevention interventions, content of training of prevention professionals, credentialling of prevention professionals and certification of prevention service providers—is also available from a variety of organizations, such as universities, the Society for Prevention Research, and the National Prevention Network. Prevention services themselves are delivered primarily by community-based organizations, such as schools, prevention providers, social, family and health agencies, and law enforcement.

A fully integrative and comprehensive system coordinates evidence-based approaches such as the one presented in Fig. 4 showing a cross sector/system collaboration of physical, behavioral, social, and health sectors that are critical to an effective plan to thwart the substance use epidemic. Our current fragmented response systems create barriers to achieving this goal. Policies that support this integration, with guidance from the Advisory Council mentioned above, will lead to fundamental changes to more effectively and equitably advance healthy outcomes and prevent development of SUD. Changing infrastructure, incentives, and funding streams to support greater collaboration and teamwork provide for a “whole-community” approach.

## Conclusion

Tackling this national substance use crisis requires broad and consistent investment in preventive strategies and an appreciation for public health approaches overall. By embracing the well-tested strategies developed over the decades by prevention scientists, communities can be supported to implement evidence-based programs that steer children away from drug use. Programs and policies of these sort need to be implemented across the life-course with special emphasis during key developmental transitions (e.g., early childhood and adolescence) to provide for a safe, nurturing environment for healthy development.

True improvements in our nation’s policies that focus on substance use require a more balanced portfolio that supports the full range of effective strategies reflective of the needs of the U.S. population, with the majority of support for prevention services, followed by tertiary approaches (e.g., treatment, regulations, harm reduction). Furthermore, the added benefits of these evidence-based prevention strategies include improved academic performance, reduced bullying and violence, and better emotional and physical health that enhance positive life courses and enhanced community participation (MacArthur et al., 2018).

Scaling and sustaining prevention strategies at scale further requires dedication of all concerned stakeholders and support from policymakers. But ultimately, it will pay off by fostering a healthier and better-connected community that prioritizes the elimination of adverse social conditions and implementing prevention science-based programs shown to both promote healthy child development and avoid wasting taxpayers’ money.

## Declarations

### Data Availability

No data were collected or reported in this manuscript.
